# Improvement of fertility outcomes using dexamethasone / antibiotic adjuvant therapy following hysterosalpingography in infertile females: A randomised trial

**DOI:** 10.12669/pjms.38.5.5432

**Published:** 2022

**Authors:** El-Kharoubi Amin-Florin, Szasz Florin

**Affiliations:** 1 El-Kharoubi Amin-Florin, PhD, Department of Obstetrics and Gynaecology, Faculty of Medicine and Pharmacy, University of Oradea, 1st December Square 10, 410068 Oradea, Romania; 2 Szasz Florin, PhD, Department of Obstetrics and Gynaecology, Faculty of Medicine and Pharmacy, University of Oradea, 1st December Square 10, 410068 Oradea, Romania

**Keywords:** Fallopian tube patency, Diagnosis, Infertility, Hysterosalpingography, Laparoscopy

## Abstract

**Objectives:**

To evaluate the benefit of modified hydrotubation with dexamethasone and antibiotics after hysterosalpingography in improving pregnancy rates in women with infertility issue.

**Methods:**

This retrospective study conducted at County Emergency Clinical Hospital Oradea, Bihor, Romania, between January 2017 and December 2019. One hundred twenty infertile females were investigated, as part of their evaluation, Hysterosalpingography was performed. After the investigation 97 patients had taken utero-tubal instillations with (Ceftriaxone 1 gr. [Cefort] and 4 mg of Dexamethasone, Lidocaine 1% 10 ml, and a Sodium chloride 0.9% 10 ml), and 23 patients were included in the control group which didn’t receive utero-tubal instillations. Number of obtained pregnancies by natural way over the next year was compared in the two groups.

**Results:**

From all participants in this study, 30 women became pregnant during the study. In addition, 29 out of 30 pregnant women had taken hydrotubation procedure. The significant statistical difference was observed between the groups p=0.011 (p<0.05), and the odd ratio was less than one (OR=9.3, 95%, CI: 1,207 to 72.926). We also found an indirect correlation between abortion in the past and the pregnancy ratio (r=-0.21).

**Conclusion:**

The results of the study demonstrated that the application of modified hydrotubation with the administration of dexamethasone and antibiotics in patients who had at least one patent fallopian tubes, can increase the chance of fertility.

## INTRODUCTION

Infertility is defined as a failure to achieve pregnancy within 12 and six months of unprotected intercourse in females younger and older than 35 years, respectively.^1^ It is estimated that around 10% of a couple of the reproductive age suffer from infertility worldwide.^2^ According to some studies, the prevalence rate of this condition reaches 15% in developed nations.^3^ In more than 35% of the infertile couple, female infertility was found to be the cause; moreover, more than 50% of the identifiable causes of female infertility were attributed to anatomical abnormalities.^4^ Furthermore, fallopian tube abnormalities are estimated to be between 25% and 35% of the reported infertility cases. Accordingly, it is of critical importance to understand the causes to successfully manage this problem. Pelvic inflammatory disease (PID) is the most common cause of tubal disease, which can lead to tubal blockage.

In the evaluation protocol of infertile couple, after the initial examination using the semen analysis, as well as the assessment of ovarian function and reserve, the next step is evaluation of tubal permeability,^5^ tubal patency can be evaluated by HSG with positive and negative predictive values of 97.2% and 66.7%,^6^ In case of unilateral tubal blockage, , some studies suggested that the success rates of intrauterine insemination(IUI) after three cycles of IUI were 26.3% and that in vitro fertilisation instead of IUI may be a more appropriate approach.^7^ It is worth mentioning that new studies and new technique contribute to the increase of success rate of infertility treatment over the years.^8^

Intraperitoneal adhesions are one serios issue in infertility, which is caused by various pathogens that can infect the female genital tract, in recent years some studies suggest the possible beneficial effect of administration of dexamethasone intraperitoneal after surgeries to decrees the risk of intraperitoneal adhesions.^9^

This study aimed to investigate whether after performing HSG for woman diagnosed with infertility, and with at least one of the tube patents, flushing the tubes with dexamethasone and antibiotics in the next days, to decrees the local inflammation and to combat the possible infection can result in higher chance of becoming pregnant by natural ways.

## METHODS

This prospective study was conducted at County Emergency Clinical Hospital Oradea, Bihor, Romania. This study investigated the medical records of one hundred and twenty infertile females (age range: 22 to 44 years) who attended the infertility clinic between January 2017 and December 2019, for treatment and underwent HSG. Moreover, the American College of Obstetricians and Gynaecologists’ definition of infertility was adopted in this study. According to this definition, infertility is the failure to achieve pregnancy within 12 and 6 months of unprotected intercourse in females younger and older than 35 years, respectively. Ethical clearance and informed consent were obtained from the participants. The study protocol was approved by the Local Research Ethics Committee at Oradea University of Medical Sciences, and the Ethics Committee County Emergency Clinical Hospital Oradea, Bihor, Romania. (Nr. 4237) all methods were performed in accordance with the relevant guidelines and regulations.

Inclusion criteria were no prior pelvic surgery and normal bimanual pelvic examination. Patients with medical disorders, contraindications for HSG, uterine abnormalities, or bilateral tubal blockage result on HSG, or patients under vitro fertilisation treatment were excluded from the study. In total, 120 patients met the inclusion criteria.

Utero-tubal installation (Ceftriaxone 1 gr. [Cefort] and 4 mg of Dexamethasone, Lidocaine 1% 10 ml, and a Sodium chloride 0.9% 10 ml) was offered as a treatment in the next day after HSG procedure. The utero-tubal flashing was optional, every patient had the opportunity to accept it or to skip it, the risk and the potential benefits were explained to every patient.

In this study we used descriptive and inferential statistics for data analysis. We presented the descriptive data in mean and standard deviation. For inferential analysis, the obtained data were analysed in IBM SPSS software (Version 25.025) through Spearman correlation and the Chi-Square test, and the results presented as average SEM (standard error). A p-value less than 0.05 was considered statistically significant.

The HSG was performed between Day-7 and 10 of the menstrual cycle with a period of 36-48 hour after menstruation. The women were advised to avoid unprotected intercourse in this period. The patients were placed in a lithotomy position, and a vaginal speculum was inserted. After cleaning the cervix and the vagina, the cervix was grasped, and a cannula was then inserted into the cervix. Approximately, 10-15 mL of a water-soluble contrast was injected manually through the cannula. The X-ray films (n=2), as well as images of early and maximal opacification of the uterine cavity, fallopian tubes, and peritoneal contrast spillage, were obtained, the readings are done by our radiologist.

The HSG was performed under general anaesthesia as a standard protocol in our unit, and the results were given the same day. If at least one of the tubes was patent, in the next day utero-tubal installation with (Ceftriaxone 1 gr. [Cefort] and 4mg of Dexamethasone, Lidocaine 1% 10 ml, and a Sodium chloride 0.9% 10 ml) was offered in local anaesthesia. This procedure is usually performed two to three times in the next two three days after HSG procedure to reduce the inflammation and keep the tube permeable.

Additionally, the following data were collected and evaluated: the results of HSG, data of utero-tubal instillation, demographic characteristics (age), as well as primary and secondary infertility, obstetrics and gynaecological history, and vaginal infection tested 10 days prior of the procedure.

All patients were follow-up by call after six months and one year from the date of the procedure. It included evaluations for presence of pregnancy in the past one year after the intervention.

## RESULTS

From the total number of 120 patients, 97 patients had taken the utero-tubal installations (Group A), and 23 were included in the control group (Group B) which are patients whom didn’t take utero-tubal instillation after HSG.

The mean±SD age of our lot of patients was 30.35±4.283 years, we classified the patients in three category by age (a= between 20-30 years old, b= between 30-40, and c= bigger the 40 years old), 64 were in “a” category, 54 were in “b” category and two were over the age of 40.

Regarding the primary and secondary infertility, in our lot 82 (68.3%) and 38 (31.7%) females had primary and secondary infertility, 30 women had given birth previously, and fourteen had an abortion in the past, and among the female whom had given birth before a number of 5 had had an abortion also in the past.

It should be mentioned that none of the patients had IVF before; additionally, they reported no other medical disorders or smoking history, Furthermore, none of the patients had an intrauterine device in the past; however, 17 patients had used oral contraceptives previously - all of them used it for prevention. No patient had a BMI of >35.

According to the results of HSG, 52 (43.3% of all the patents included in our study) had bilateral tubal patency and 68 (56.7%) patients had unilateral patency. The mean age of the patients with bilateral patency was obtained at 30.50±3.8.

Genital tract infections were tested 10 days before the procedure, a number of 62 (51.67%) females had a vaginal infection, the pathogens found were: Coccobacillus (n=30; 25.0%), Candida (n=11; 9.16%), and Gardnerella (n=10; 8.33%). Moreover, staphylococcus H, E-coli, and Trichomonas vaginalis were observed in 3 (2.5%), 5 (4.16%), and 1(0.83%) patient.

**Chart-1:**
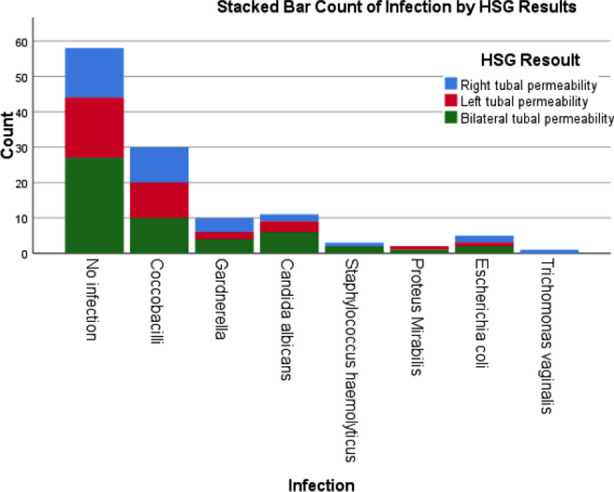
Types of vaginal infection by the HSG results.

In total, 25 cases of those with bilateral permeability had a vaginal infection. The type of germ found in the laboratory examination is presented in (Chart-1). A correlation was searched between vaginal infection detected on the examination of vaginal discharge and the HSG result of tubal patency, no correlation was found in our results. Also, no correlation was found between the results of HSG and primary and secondary infertility.

Regarding the follow-up, the females were followed-up using a telephone, 12 months after the procedure. The questions focused primarily on infertility issues and whether the intrauterine pregnancy was achieved by natural conception.

### Rate of pregnancies and treatment group:

According to our results, a total of 30 (25%) females got pregnant of which a number of 14 of them got pregnant in the first 6 month after the procedure, and a number of 16 in the first year.

Of our study group (the patients whom got utero-tubal instillation) 29 females out of 97 of them got pregnant, and just one out of 23 of the controlled group archived pregnancy. The chance of getting pregnant was higher in patients who were in the age range of 20-30 years with the mean age of 29.17 years, succeeded by those in the age range of 30-40 years (Table-I). 10 females who got pregnant after the utero-tubal installation were those who had given birth before, and none of them had an abortion in the past.

**Table I:** Age distribution of the patients and infertility type and the outcome.

	Primary Infertility				Secondary infertility				

	Age distribution				Age distribution				

	20-30	Age 31-40	Age >41	Total	20-30	Age 31-40	Age >41	Total	
	Count	Count	Count	Count	Count	Count	Count	Count	
Patients whom got pregnant	No	31	30	1	62	13	15	0	28
	Yes	15	4	1	20	5	5	0	10

However, we corelate the result of achieving pregnancy and our groups of study (Group-A vs Group B) and the result was a *p*-value was at 0.011, (which was smaller than the standard alpha value (P<0.05)); therefore, the null hypothesis which indicated the independence of two variables was rejected in this study. In other words, there was a direct correlation (r=0.232) between getting pregnant and undergoing the utero-tubal installation (Odds ratio [OR] = 9.3, 95% CI 1.207-72.926).

The correlation of some variables with pregnancy was also investigated in this study (Table-II). A negative correlation was observed between abortion in the past and pregnancy ratio (r= -0.21). On the other hand, there was no significant correlation between the infection and pregnancy outcome within 12 months after the installation.

**Table II:** Correlation between the various variables of the patients.

		Patients whom got pregnant	Patients with vaginal pathogens	Abortion in the past	Patents with childbirth in the past
Patients whom got pregnant	Pearson Correlation	1	.087	-.210^*^	.099
	Sig. (2-tailed)		.343	.021	.282
	N	120	120	120	120
Patients with vaginal pathogens	Pearson Correlation	.087	1	.007	-.031
	Sig. (2-tailed)	.343		.941	.738
	N	120	120	120	120
Abortion in the past	Pearson Correlation	-.210^*^	.007	1	.201^*^
	Sig. (2-tailed)	.021	.941		.028
	N	120	120	120	120
Patents with childbirth in the past	Pearson Correlation	.099	-.031	.201^*^	1
	Sig. (2-tailed)	.282	.738	.028	
	N	120	120	120	120

* Correlation is significant at the 0.05 level (2-tailed).

## DISCUSSION

The mean age of the patients was 30.35±4.2 years in this study. The delays in pregnancy may be due to studying and pursuing a professional career by females. Accordingly, they got pregnant at older ages when oocyte number and quality decrease. Based on the Eurostat data, this trend is similar to that in European countries regarding delays in pregnancy. The infertility rate increases with increasing age, which is in line with the results of previously conducted studies that have reported lower fertility rates in older females.^7^,^10^

This study attempted to search for a correlation between various paraclinical factors, such as vaginal infection, and the HSG results. A number of 62 (51.67%) females who were referred to our infertility department had a genital tract infection, which was reported to affect the reproductive outcome.^11^ The pelvic inflammatory disease is the most common cause of tubal disease, which is originated from genital infection, especially Chlamydia trachomatis and Neisseria gonorrhoeae, Recent studies shows the increase of tubal cause infertility by tubal fibrosis in patients with chlamydia antibodies present.^12^ In the present study, no correlation was found between the HSG results and specific pathogens causing genital tract infections, and the vaginal discharge which was tested 10 days before the procedure. But we have to mention that we limit our study on present infections, and no antibodies were tested. Regarding the increased age of the study group, there is a possibility that an asymptomatic or symptomatic infection in the past. Also, a new study found the correlation between chlamydia antibodies and tubal patency which shows a strong correlation between presence of antibodies and bilateral obstruction, and no significant association between presence of antibodies and unilateral tubal obstruction.^13^

Totally, 120 females were included in this study, the majority of whom (n=97) were given utero-tubal installation after HSG. An important and expected result in the current study was the correlation between the patients who received utero-tubal installation and those who did not receive it. Those who underwent this procedure got a higher chance of becoming pregnant.

During the first 12 months after the procedure, 30 patients became pregnant, one of whom was in the category of those who did not receive utero-tubal installation. A possible explanation of the results is an effect on the tubal spasm and the anti-inflammatory effect of dexamethasone on the tubes. Regarding the correlation between the past gestation status of the patients and the fertility outcome, a higher chance of fertility was observed in females with a previous birth; however, it showed no direct relationship between vaginal infections and fertility results.

According to a study conducted by Irving I. Kurland, intramuscularly administered dexamethasone had a beneficial effect on tubal patency within six months of the administration.^11^ In the same line, some studies suggest the beneficial effect of dexamethasone on ovulation.^14^,^15^ Another study compared the pregnancy rate in tow groups with inducing ovulation with letrozole alone comparing with letrozole plus dexamethasone and the result show an increase in pregnancy rate for those treated with letrozole plus dexamethasone.^16^

There were no other studies to make a comparison between their findings and the results obtained from this study; however, some studies compared flushing the tubes with oil-soluble contrast media versus no treatment on 204 females with a 3.27, 95% CI 1.57 to 6.85, the meta-analysis of 14 RCTs reveal that tubal flushing using oil-based contrast likely enhance short-term (six months) fertility rate in unexplained infertility^17^ In comparison to our study from 14 patients which represent nearly half of the patients who got pregnant in the first six month and the rest of 16 got pregnant in the period of six month to one year. As regards the beneficial effect of tubal flushing there are several hypotheses on the possible mechanism, but no good-quality evidence on the therapeutic effect of these tests studies are available.^18^

Another recent study shows the beneficial of intrauterine perfusion with dexamethasone downregulation of the proportion of Natural-Killer cells, which improve endometrial receptivity and enhance embryo implantation.^19^

Hysterosalpingography remains valuable diagnostic tool even through its diagnostic value is inferior in comparison to laparoscopy,^20^ and tubal flushing is a simple manoeuvre, which can be done easily by gynaecologist. Our research result show that it increases the chance of achieving pregnancy, the utero-tubal flushing can be a standard recommendation for patients who undergo hysterosalpingography and the result shows at least a patent tube, can also be a recommendation after laparoscopy for fertility issues.

### Limitation of the study:

Regarding the limitations of the study, one can name the small number of the study population; therefore, further studies are recommended to be conducted using a larger sample size in this regard.

## CONCLUSION

This study indicates that performing utero-tubal instillation with dexamethasone can improve the outcome of the fertility with minimum to no harm for the patient. More well-designed studies are needed to confirm our observaitons and further validate the effect of utero tubal instillation on female fertility outcomes.
